# BNT162b2 Vaccine Encoding the SARS-CoV-2 P2 S Protects Transgenic hACE2 Mice against COVID-19

**DOI:** 10.3390/vaccines9040324

**Published:** 2021-04-01

**Authors:** Rui-Ru Ji, Yajin Qu, Hua Zhu, Yumei Yang, Annette B. Vogel, Ugur Sahin, Chuan Qin, Aimin Hui

**Affiliations:** 1 Shanghai Fosun Pharmaceutical Industrial Development, Co. Ltd., 1289 Yishan Road, Shanghai 200233, China; ruiruji@msn.com (R.-R.J.); yangyumei@fosunpharma.com (Y.Y.); 2 NHC Key Laboratory of Human Disease Comparative Medicine, Beijing Key Laboratory for Animal Models of Emerging and Remerging Infectious Diseases, Institute of Laboratory Animal Science, Chinese Academy of Medical Sciences and Comparative Medicine Center, Peking Union Medical College, Beijing 100021, China; quyajin@pumc.edu.cn (Y.Q.); zhuhua@pumc.edu.cn (H.Z.); 3 BioNTech, An der Goldgrube 12, 55131 Mainz, Germany; Annette.Vogel@biontech.de (A.B.V.); Ugur.Sahin@biontech.de (U.S.); 4 TRON gGmbH—Translational Oncology at the University Medical Centre of the Johannes Gutenberg University, Freiligrathstraße 12, 55131 Mainz, Germany

**Keywords:** SARS-CoV-2, COVID-19, mRNA vaccine, efficacy, immunogenicity, challenge study

## Abstract

BNT162b2 is a highly efficacious mRNA vaccine approved to prevent COVID-19. This brief report describes the immunogenicity and anti-viral protective effect of BNT162b2 in hACE2 transgenic mice. Prime-boost immunization with BNT162b2 elicited high titers in neutralizing antibodies against SARS-CoV-2, which correlated with viral clearance and alleviated lung lesions in these mice after viral challenge.

## 1. Introduction

SARS-CoV-2 infects cells through the binding of the spike (S) viral membrane protein to the human angiotensin converting enzyme 2 (hACE2) on target cells [1]. A transgenic C57BL/6 mouse, expressing the hACE2 gene under the control of the mouse Ace2 promoter, was first developed as a model system to study severe acute respiratory syndrome (SARS) caused by SARS-CoV [2]. Using the hACE2 transgenic mouse model, SARS-CoV-2 replication was observed in the lungs of infected mice, and viral antigens were detected in bronchial epithelial cells, macrophages, and alveolar epithelia. The typical histopathology was interstitial pneumonia with infiltration of considerable numbers of macrophages and lymphocytes into the alveolar interstitium, and the accumulation of macrophages in alveolar cavities. By contrast, wildtype mice challenged with SARS-CoV-2 did not develop these symptoms [3]. These data suggested that the hACE2 transgenic mouse is a valuable model for the evaluation of vaccines and antiviral therapeutic agents fighting COVID-19. 

BNT162b2 is a lipid nanoparticle (LNP) formulated nucleoside-modified mRNA vaccine expressing the full-length S protein stabilized in the prefusion conformation (P2 S, containing K986P and V987P) [4,5,6]. BNT162b2 is being tested in several clinical trials including global Phase 2/3 trials (NCT04380701, NCT04368728) to investigate its safety, immunogenicity, and efficacy in humans [7,8]. Until 16th December 2020, BNT162b2 has received 16 emergency approvals. Our previous preclinical studies demonstrated that immunization of wildtype BALB/c mice with a single dose of BNT162b2 intramuscularly induced a fast and dose-dependent increase in total IgG response and pseudo-virus neutralization titers. In rhesus macaques, two vaccinations elicited high SARS-CoV-2 neutralization titers. In both animal models, a strong T helper type 1 (Th1) bias with a high IFNγ^+^ CD8^+^ T-cell response was observed, and BNT162b2 fully protected the lungs of immunized rhesus macaques from the SARS-CoV-2 challenge [9]. Addressing the limited utility of rhesus macaques as a disease model, we report the preclinical efficacy evaluation of BNT162b2 in a challenge study using hACE2 transgenic mice. 

## 2. Materials and Methods

### 2.1. Ethics Statement

This mouse study was performed in an animal biosafety level 3 (ABSL3) facility using HEPA-filtered isolators. All procedures in this study involving mice were reviewed and approved by the Institutional Animal Care and Use Committee of the Institute of Laboratory Animal Science, Peking Union Medical College (GH20012). All experiments complied with all relevant ethical regulations.

### 2.2. Cell lines and Virus

The human Vero E6 cell line was cultured in Dulbecco’s modified Eagle’s medium (DMEM; Invitrogen, Carlsbad, CA, USA) supplemented with ten percent fetal bovine serum (Gibco, Grand Island, NE, USA) and incubated at 37 °C and five percent carbon dioxide. The SARS-CoV-2 isolate SARS-CoV-2/human/CHN/WH-09/2020 (GenBank: MT093631.2) [10] was used. 

### 2.3. Construction of the BNT162b2 Vaccine

BNT162b2 was generated as described elsewhere (Vogel et al., 2020). Briefly, a full-length SARS-CoV-2 spike protein (GenBank: MN908947) including the K986P and V987P mutations was designed (P2 S) [4,5,6]. In vitro transcription was analyzed using T7 RNA polymerase in the presence of a trinucleotide cap1 analogue ((m_2_^7,3′-O^)Gppp(m^2′-O^)ApG; TriLink, San Diego, CA, USA) and with N1-methylpseudouridine-5′-triphosphate (m1ΨTP; Thermo Fisher Scientific, Schwerte, Germany) replacing uridine-5′-triphosphate (UTP) [11]. The purified RNA was formulated into lipid nanoparticles (LNPs) using an ethanolic lipid mixture of ionizable cationic lipid [12]. The vaccine candidate was stored at −70 to −80 °C at a concentration of 0.5 mg/mL.

### 2.4. Study Design Animal Experiments

Mouse studies were performed in an animal biosafety level 3 (ABSL3) facility using HEPA-filtered isolators. Table 1 contains the animal grouping and testing information, and Supplemental Figure S1 shows a schematic view of the study design. C57BL/6 female and male hACE2 knockin mice [3] approximately 6 weeks of age at study start were randomly assigned to three groups: control (injection dilution buffer), medium dose (1 µg/animal), and high dose (5 µg/animal). The animal body weights were 20–23 g.

Routine animal monitoring such as body weight and macroscopic assessment of animal activity and behavior was carried out daily throughout the study period. Primary and second immunizations occurred on day 0 and day 21 via intramuscular injection (i.m.) at an injection volume of 20 µL per injection per animal. On day 28 blood samples were collected from all mice, and sera of every 2~3 mice in each group were mixed to detect total IgG and neutralizing antibodies. 

After being intraperitoneally anaesthetized by 2.5% Avertin (tribromoethanol) with 0.02 mL/g body weight, mice were challenged intranasally on day 42 with 50 µL viral suspension of strain SARS-CoV-2/WH-09/human/2020/CHN at 10^5^ TCID_50_ per animal. The mice were observed continuously for 5 days after challenge, and the weight changes were recorded. Five days after challenge (day 47), all mice were sacrificed for viral load detection and pathological examination of lung tissues.

### 2.5. S1 Binding IgG Assay

The 96-well plates were coated with recombinant S1 (100 ng/100 µL, Sino Biological, Beijing, China) in sodium carbonate buffer, and bound IgG was detected using an HRP-conjugated secondary antibody (Jackson ImmunoResearch Laboratories, Cambridge, UK) and TMB substrate (Sino Biological, Beijing, China). Data collection was performed using a Multiskan MK3 reader (Thermo Fisher, Waltham, MA, USA). The OD value (450–630 nm) was calculated.

### 2.6. SARS-CoV-2 Virus Neutralisation Test

SARS-CoV-2 strain SARS-CoV-2/human/CHN/WH-09/2020 (GenBank: MT093631.2) was used in the virus neutralization test (VNT), and the serum samples were incubated at 56 °C for 30 min for thermal inactivation. Dulbecco’s modified Eagle’s medium (DMEM) was used to continuously dilute each serum sample. The dilution ratio was 2 or 3 times, depending on OD value or sample quantity. The staring dilution was 1:8 for BNT162b2 sera. Serum dilution was mixed with the same volume of diluted virus and incubated at 37 °C for 1 h. The Vero E6 cells in the 24-well plate were incubated with the serum virus mixture at 37 °C. After 1 h, DMEM containing 2.5% FBS and 0.8% carboxymethyl cellulose was used to replace the mixed culture medium of serum virus in the wells. They were fixed with 8% paraformaldehyde and dyed with 0.5% crystal violet 3 days later. All samples were repeated, and the neutralization titer was defined as a serum dilution ratio that resulted in a reduction in plaque by at least 50%.

### 2.7. RNA Extraction and Reverse-Transcription Quantitative Polymerase Chain Reaction

The virus load was analyzed by RT-qPCR. Total RNA was extracted from organs using the RNeasy Mini Kit (Qiagen, Hilden, Germany), and reverse transcription was performed using the PrimerScript RT Reagent 203 Kit (TaKaRa, Kusatsu, Japan) following the manufacturer instructions. Quantitative real-time reverse transcription-PCR (qRT-PCR) reactions were performed using the PowerUp SYBG Green Master Mix Kit (Applied Biosystems, USA), in which samples were processed in duplicate using the following cycling protocol: 50 °C for 2 min, 95 °C for 2 min, followed by 40 cycles at 95 °C for 15 s and 60 °C for 30 s, and then 95 °C for 15 s, 60 °C for 1 min, and 95 °C for 45 s. The primer sequences used for qRT-PCR are targeted against the envelope (*E*) gene of SARS-CoV-2 and are as follows: forward: 5′-TCGTTTCGGAAGAGACAGGT-3′; reverse: 5′-GCGCAGTAAGGATGGCTAGT-3′. The PCR products were verified by sequencing using the dideoxy method on an ABI 3730 DNA sequencer (Applied Biosystems, Foster City, CA, USA). During the sequencing process, amplification was performed using specific primers. The sequencing reads obtained were linked using DNAMAN, and the results were compared using the Megalign module in the DNAStar software package. The SYBR green real-time PCR standard curve was generated by serial tenfold dilutions of recombinant plasmid with a known copy number (from 1.47 × 10^9^ to 1.47 × 10^1^ copies per μL). These dilutions were tested and used as quantification standards to construct the standard curve by plotting the plasmid copy number against the corresponding threshold cycle values (Ct). Results were expressed as log10-transformed numbers of genome equivalent copies per mL of sample.

### 2.8. Histopathology

Autopsies were performed in an animal biosafety level 3 (ABSL3) laboratory. Major organs were grossly examined and then fixed in 10% buffered formalin solution.

The lung tissue was fixed in 10% buffered formalin solution, and paraffin sections (3–4 μm in thickness) were prepared routinely. Hematoxylin and Eosin (H&E) stain was used to identify histopathological changes. The histopathology of the lung tissue was observed by light microscopy and scored based on the severity of lesions (thickening of alveolar septum and infiltration of inflammatory cells, and perivascular inflammatory cell infiltration). 

### 2.9. Statistical Analysis

All data were analyzed with GraphPad Prism 8.0 software. Statistically significant differences between two groups were determined using unpaired Student’s *t*-tests. A two-sided *p* < 0.05 was considered to be statistically significant.

## 3. Results and Discussion

Transgenic hACE2 mice were immunized twice, three weeks apart, with either a medium (1 µg) or high (5 µg) dose of BNT162b2 or dilution buffer (control) (Table 1 and see Supplementary Materials, Figure S1). During the immunization period and prior to virus challenge, the animals in all groups had similar normal food and water intake and steady weight gain. No clinically significant abnormal findings were observed. 

**Figure 1:**
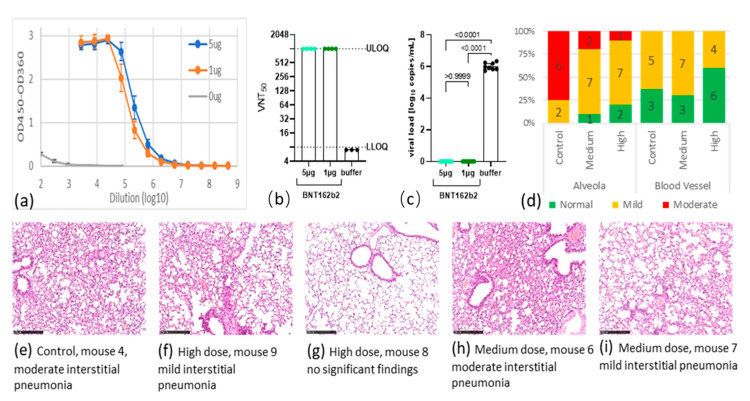
Antibody immune response after BNT162b2 immunization (a+b) and analysis of protection after viral challenge (**c**–**i**). (**a**) Titration of anti-S1 IgG antibodies analysis by enzyme-linked immunosorbent assay (ELISA); (**b**) titers of 50% virus-neutralizing antibodies; (**c**) viral load in lung tissue and associated pairwise *p* values 5 days after challenge; (**d**) percentage (y axis) and number (data labels) of mice grouped by lung symptom and dose group. Lung symptom was scored as normal (green), mild lesion (orange), or moderate lesion (red), for alveolar septum (left) and tissue around blood vessels (right); (**e**–**i**) representative Hematoxylin and Eosin (H&E) stained lung tissue slices used to identify histopathological changes. Scalebar is 250 μm, HE ×100. Classification criteria for alveolar septum: mild lesion—mild thickening of the alveolar septum; moderate lesion—obvious thickening of the alveolar septum, with the lesion range greater than ½. Classification criteria of inflammatory cells infiltration around blood vessels: mild lesion—lesion range less than 1/4 of the lung tissue section; moderate lesion—lesion range being 1/4 to 2/4 of the lung tissue section.

Seven days after the second immunization (day 28), mice were bled to quantify S1 specific antibodies. BNT162b2 was able to induce high serum IgG titers in immunized mice also using 1 µg (Figure 1a). The levels of neutralizing antibody in the BNT162b2 group all reached the upper limit of quantification (ULOQ) at a titer of 1024 (Figure 1b). By contrast, the control group had no detectable anti-S1 IgG binding nor neutralizing antibodies. 

Three weeks after the second immunization (day 42), animals were challenged intranasally with 10^5^ TCID_50_ SARS-CoV-2, continuously observed for five days, then sacrificed for final analysis of viral load and histopathology of lung tissues. After virus challenge, every experimental group experienced a drop in body weight (see Supplementary Materials, Table S1). The weight losses were 6.58% in the control, 5.57% in the 1 µg, and 7.88% in the 5 µg dosed groups. There was no statistically significant difference in weight loss among the groups (*p* > 0.05). Five days after virus challenge, the mean viral load in the control group was 10^6.01^ copies/mL. By contrast, no viral RNA could be detected in the lung tissues in BNT162b2-treated mice, suggesting that viral replication was completely inhibited in those animals (Figure 1c and see Supplementary Materials, Table S2). Taken together with the virus neutralization test (VNT) data, the complete suppression of viral replication coincided with the high titer of neutralizing antibodies in the mice.

Scoring of lung lesions of these mice is summarized in Figure 1d (see Supplementary Materials, Table S3), and representative staining of lung tissues from every group are shown in Figure 1e–i. After viral challenge, all hACE2 mice in the control group developed mild or moderate interstitial pneumonia, characterized by thickening of alveolar septum and infiltration of inflammatory cells in the lungs. BNT162b2 vaccination at both doses ameliorated these lung lesions. For example, two animals in the medium-dose group developed moderate thickening of alveolar septum and infiltration of inflammatory cells, seven had mild phenotype, and one had no lesion. The high-dose group showed further improvement: two were free of any lesions, seven had mild, and one had moderate lesions (Figure 1d). 

Although we have observed improved lung lesions in vaccinated groups, the phenotypic manifestations of virus infection were variable among these animals and not directly correlated with the clearance of the virus or neutralizing antibody titers. For example, BNT162b2 vaccination cleared the virus completely in the lungs; however, these mice still exhibited various degrees of lung lesions. The lack of a strong correlation among these measures such as viral load, weight changes, and lung lesions limits our ability to rationalize the observations. 

There are clear limitations to this mouse model, such as the limited tissue distribution of hACE2 expression and a relatively mild histopathology [2]. A very different approach using hACE2-transduced wildtype mice [13] also showed that mouse models of the disease, while critical tools, have significant limitations as a means to assess the full spectrum of the potential protective effect of vaccines and therapeutic agents.

These results support the decision to move BNT162b2 toward market authorization.

## 4. Conclusions

The reported hACE2-transduced mouse study using BNT162b2 for vaccination demonstrates that this market authorized COVID-19 vaccine is highly immunogenic and an effective mean for anti-viral protection. 

## Figures and Tables

**Table 1:** Summary of study key parameters and results.

Dose Group.	Number of Mice	Dose Per Mouse	Infection	Neutral. Ab Titer	Viral Load Log_10_	Alveolar Score			Blood Vessel Score		
						Normal	Mild	Mod.	Normal	Mild	Mod.
High	10	5 µg	10^5^TCID_50_/50 µL	1024 *	0+/−0 *	2	7	1	6	4	0
Medium	10	1 µg		>1024 *	0+/−0 *	1	7	2	3	7	0
Control	8	-		<8	6+/−0.22	0	2	6	3	5	0

* *p* << 0.001 compared to control. Listed from left to right are, for each dose group, number of animals per group; vaccine dose per animal per shot; viral infection dose per animal; titers of neutralizing antibodies with <8 being below the lower limit and >1024 being higher than upper limit of quantification; viral load given as log10 of the viral particle count; and the number of animals per lung lesion score (see Figure 1 for definition of scores).

## Data Availability

The authors declare that the data supporting the findings of this study are available within the paper and its Supplementary Information file, or available from the corresponding author(s) upon reasonable request.

## Supplementary Materials

The following are available online at https://www.mdpi.com/article/10.3390/vaccines9040324/s1, Figure S1: Schematic of study design and timelines, Table S1: BNT162b2: animal body weight in each animal after challenge, Table S2: Viral Load in lung tissue 5 days after viral challenge, Table S3: Scoring of pathological changes of lung tissue in every animal 5 days after challenge.

## References

[1] Zhou P., Yang X.-L., Wang X.-G., Hu B., Zhang L., Zhang W., Si H.-R., Zhu Y., Li B., Huang C.-L. (2020). A pneumonia outbreak associated with a new coronavirus of probable bat origin. Nature.

[2] Yang X.-H., Deng W., Tong Z., Liu Y.-X., Zhang L.-F., Zhu H., Gao H., Huang L., Liu Y.-L., Ma C.-M. (2007). Mice transgenic for human angiotensin-converting enzyme 2 provide a model for SARS coronavirus infection. Comp. Med..

[3] Bao L., Deng W., Huang B., Gao H., Liu J., Ren L., Wei Q., Yu P., Xu Y., Qi F. (2020). The pathogenicity of SARS-CoV-2 in hACE2 transgenic mice. Nature.

[4] Kirchdoerfer R.N., Wang N., Pallesen J., Wrapp D., Turner H.L., Cottrell C.A., Corbett K.S., Graham B.S., McLellan J.S., Ward A.B. (2018). Stabilized coronavirus spikes are resistant to conformational changes induced by receptor recognition or proteolysis. Sci. Rep..

[5] Pallesen J., Wang N., Corbett K.S., Wrapp D., Kirchdoerfer R.N., Turner H.L., Cottrell C.A., Becker M.M., Wang L., Shi W. (2017). Immunogenicity and structures of a rationally designed prefusion MERS-CoV spike antigen. Proc. Natl. Acad. Sci. USA.

[6] Wrapp D., Wang N., Corbett K.S., Goldsmith J.A., Hsieh C.-L., Abiona O., Graham B.S., McLellan J.S. (2020). Cryo-EM structure of the 2019-nCoV spike in the prefusion conformation. Science.

[7] Walsh E.E., Frenck Jr R.W., Falsey A.R., Kitchin N., Absalon J., Gurtman A., Lockhart S., Neuzil K., Mulligan M.J., Bailey R. (2020). Safety and Immunogenicity of Two RNA-Based Covid-19 Vaccine Candidates. N. Engl. J. Med..

[8] Polack F.P., Thomas S.J., Kitchin N., Absalon J., Gurtman A., Lockhart S., Perez J.L., Marc G.P., Moreira E.D., Zerbini C. (2020). Safety and Efficacy of the BNT162b2 mRNA Covid-19 Vaccine. N. Engl. J. Med..

[9] Vogel A.B., Kanevsky I., Che Y., Swanson K.A., Muik A., Vormehr M., Kranz L.M., Walzer K.C., Hein S., Güler A. (2021). BNT162b vaccines protect rhesus macaques from SARS-CoV-2. Nature.

[10] Deng W., Bao L., Liu J., Xiao C., Liu J., Xue J., Lv Q., Qi F., Gao H., Yu P. (2020). Primary exposure to SARS-CoV-2 protects against reinfection in rhesus macaques. Science.

[11] Grudzien-Nogalska E., Kowalska J., Su W., Kuhn A.N., Slepenkov S.V., Darzynkiewicz E., Sahin U., Jemielity J., Rhoads R.E. (2013). Synthetic mRNAs with superior translation and stability properties. Methods Mol. Biol..

[12] Maier M.A., Jayaraman M., Matsuda S., Liu J., Barros S., Querbes W., Tam Y.K., Ansell S.M., Kumar V., Qin J. (2013). Biodegradable lipids enabling rapidly eliminated lipid nanoparticles for systemic delivery of RNAi therapeutics. Mol. Ther..

[13] Hassan A.O., Case J.B., Winkler E.S., Thackray L.B., Kafai N.M., Bailey A.L., McCune B.T., Fox J.M., Chen R.E., Alsoussi W.B. (2020). A SARS-CoV-2 Infection Model in Mice Demonstrates Protection by Neutralizing Antibodies. Cell.

