# Supplementary material for: BNT162b2 Vaccine Encoding the SARS-CoV-2 P2 S Protects Transgenic hACE2 Mice against COVID-19
Source: Vaccines (Basel). 2021 Apr 1;9(4):324. doi: 10.3390/vaccines9040324 (PMC8066210; doi:10.3390/vaccines9040324)
Supplement: Supplementary file 1 [file vaccines-09-00324-s001.pdf]

Supplementary data

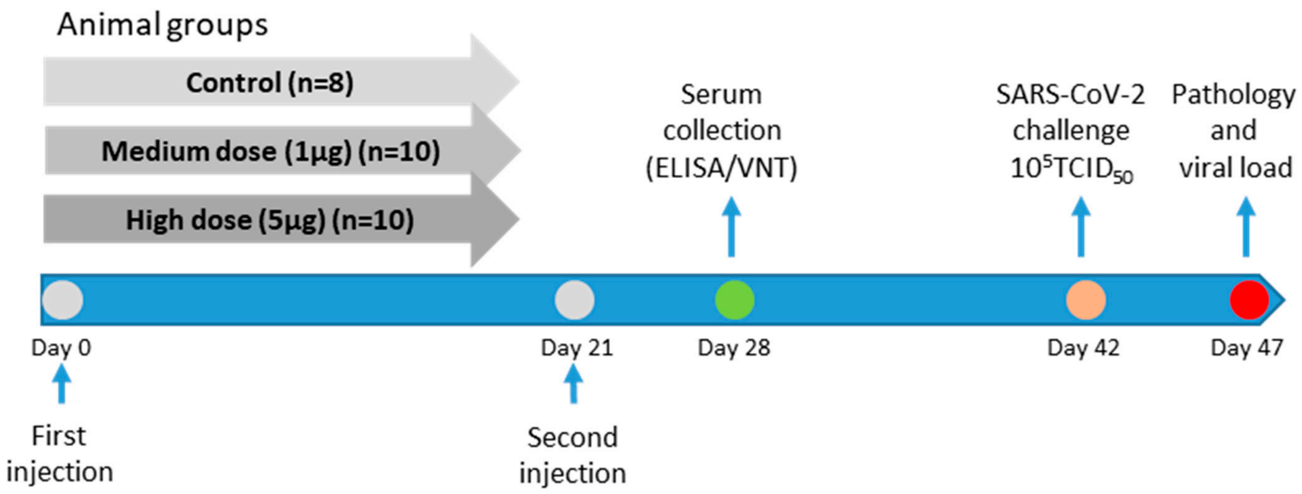

Figure S1: Schematic of study design and timelines

Table S1. BNT162b2: animal body weight in each animal after challenge

BNT162b2 Body Weight (g) for the five days (days 42 to 47) after virus challenge							
Animal group	No./Day	42	43	44	45	46	47
High	1	29.02	28.05	27.12	26.83	26.07	26.26
High	2	29.12	28.44	27.52	27.55	27.03	27
High	3	29.12	28.39	27.66	27.64	27.47	27.39
High	4	28.68	28.46	27.72	27.38	26.65	26.54
High	5	28.56	28.25	27.63	27.25	27.01	26.86
High	6	28.95	28.47	27.86	27.66	26.16	26.04
High	7	29.29	28.59	27.95	27.61	26.82	26.68
High	8	28.96	28.05	27.12	27.04	26.49	26.35
High	9	29.02	28.67	28.06	28.01	27.58	27.46
High	10	29.71	29.05	28.03	27.21	27.01	26.94
Medium	1	29.53	29.03	28.78	28.51	28.15	27.87
Medium	2	29.43	29.26	28.92	28.62	28.65	28.64
Medium	3	28.35	28.17	27.96	27.79	27.39	27.21
Medium	4	28.56	27.75	27.49	27.45	27.03	26.85
Medium	5	28.59	27.72	27.46	27.18	26.94	26.71
Medium	6	29.46	29.15	28.86	28.58	28.01	27.62
Medium	7	29.66	28.81	28.46	28.22	28.04	27.72
Medium	8	29.02	28.41	27.93	27.65	27.14	26.65
Medium	9	29.12	28.72	28.54	28.38	27.97	27.87
Medium	10	29.34	28.42	28.22	27.96	27.76	27.71
Control	1	29.24	29.27	28.72	28.47	28.25	28.02
Control	2	29.84	29.77	29.12	28.72	28.63	28.45
Control	3	28.18	28.11	27.82	27.05	26.71	26.36
Control	4	29.02	28.71	28.03	27.33	26.93	26.69
Control	5	29.35	29.16	28.52	28.22	27.65	27.52
Control	6	29.14	28.79	28.15	27.69	27.24	27.07
Control	7	28.66	28.38	27.98	27.35	26.81	26.46
Control	8	28.65	28.36	27.57	26.98	26.45	26.26
High	Average±Std	29±0.32	28.4±0.3	27.7±0.34	27.4±0.35	26.8±0.5	26.8±0.47
Medium		29.1±0.46	28.5±0.55	28.3±0.54	28±0.51	27.7±0.56	27.5±0.63
Control		29±0.51	28.8±0.55	28.2±0.51	27.7±0.67	27.3±0.78	27.1±0.82

Table S2: Viral Load in lung tissue 5 days after viral challenge, expressed as $\log_{10}$(viral copies)/ml for each animal in each of the three dose groups. *: $p << 0.001$ against Control

Animal Nr/Group	High	Medium	Control
1	0	0	5.73
2	0	0	5.83
3	0	0	5.92
4	0	0	6.24
5	0	0	5.84
6	0	0	5.99
7	0	0	6.19
8	0	0	6.34
9	0	0	
10	0	0	
Ave.±Std	0.±0.0*	0±0.0*	6.0±0.22

Table S3: Scoring of pathological changes of lung tissue in every animal 5 days after challenge. Columns are the dose group (Group), animal number (No.), score of thickening of alveolar septum and infiltration of inflammatory cells (Alveola), and score for infiltration of inflammatory cells around the blood vessels (Blood Vessel).

**Animal**	**High**		**Medium**		**Control**	
NO	Alveola	Blood Vessel	Alveola	Blood Vessel	Alveola	Blood Vessel
1	++	+	++	+	++	+
2	+	-	+	-	++	+
3	+	-	+	+	++	+
4	+	-	+	+	++	+
5	-	-	+	+	+	-
6	+	+	++	+	+	-
7	+	-	+	+	++	-
8	-	-	+	-	++	+
9	+	+	+	+		
10	+	+	-	-		

Lesion classification criteria for alveolar septum:

+, Mild lesion, mild thickening of the alveolar septum;

++, Moderate lesion, obvious thickening of the alveolar septum, with the lesion range greater than 1/2;

+++, Severe lesion, obvious thickening of the alveolar septum, with thickening and fusion of the alveolar septum, obvious narrowing of the alveolar space and the lesion range greater than 1/2;

++++, Extremely severe lesion, with thickening and fusion of the alveolar septum, obvious narrowing of the alveolar space or even disappearance, local pulmonary parenchymal lesions and the lesion range greater than 3/4;

Lesion classification for blood vessels and perivascular inflammatory cell infiltration:

+, Mild lesion, with the lesion range less than 1/4 of the lung tissue section;

++, Moderate lesions, with the lesion range being 1/4 to 2/4 of the lung tissue section;

+++, Severe lesions, with the lesion range being 2/4 to 3/4 of the lung tissue section;

++++, Extremely severe lesion, with the lesion range greater than 3/4 of the lung tissue section.
